# The multifaceted roles of antibiotics and antibiotic resistance in nature

**DOI:** 10.3389/fmicb.2013.00047

**Published:** 2013-03-12

**Authors:** Saswati Sengupta, Madhab K. Chattopadhyay, Hans-Peter Grossart

**Affiliations:** ^1^IndependentHyderabad, India; ^2^Centre for Cellular and Molecular Biology (Council of Scientific and Industrial Research)Hyderabad, India; ^3^Limnology of Stratified Lakes, Leibniz Institute of Freshwater Ecology and Inland FisheriesStechlin, Germany; ^4^Institute for Biochemistry and Biology, Potsdam UniversityPotsdam, Germany

**Keywords:** antibiotics, sub-inhibitory concentration, quorum sensing, virulence, stress response, antibiotic resistance, antibiotic paradox

## Abstract

Antibiotics are chemotherapeutic agents, which have been a very powerful tool in the clinical management of bacterial diseases since the 1940s. However, benefits offered by these magic bullets have been substantially lost in subsequent days following the widespread emergence and dissemination of antibiotic-resistant strains. While it is obvious that excessive and imprudent use of antibiotics significantly contributes to the emergence of resistant strains, antibiotic resistance is also observed in natural bacteria of remote places unlikely to be impacted by human intervention. Both antibiotic biosynthetic genes and resistance-conferring genes have been known to evolve billions of years ago, long before clinical use of antibiotics. Hence it appears that antibiotics and antibiotics resistance determinants have some other roles in nature, which often elude our attention because of overemphasis on the therapeutic importance of antibiotics and the crisis imposed by the antibiotic resistance in pathogens. In the natural milieu, antibiotics are often found to be present in sub-inhibitory concentrations acting as signaling molecules supporting the process of quorum sensing and biofilm formation. They also play an important role in the production of virulence factors and influence host–parasite interactions (e.g., phagocytosis, adherence to the target cell, and so on). The evolutionary and ecological aspects of antibiotics and antibiotic resistance in the naturally occurring microbial community are little understood. Therefore, the actual role of antibiotics in nature warrants in-depth investigations. Studies on such an intriguing behavior of the microorganisms promise insight into the intricacies of the microbial physiology and are likely to provide some lead in controlling the emergence and subsequent dissemination of antibiotic resistance. This article highlights some of the recent findings on the role of antibiotics and the genes that confer resistance to antibiotics in nature.

## INTRODUCTION

The term “antibiotics” was first coined by the American microbiologist Selman Waksman and his colleagues to describe chemical substances produced by microorganisms and having antagonistic effects on the growth of other microorganisms. It excluded synthetic antimicrobials (sulfur drugs) and biological products of non-microbial origin having antagonistic effects on bacteria. Though antibiotics were introduced into the clinical practice only in the middle of the last century, the use of microorganisms for the management of microbial infections in ancient Egypt, Greece, China, and some other places of the world is well-documented. The modern era of antibiotics started with the serendipitous discovery of penicillin from the culture filtrate of a fungus, *Penicillium notatum* by Alexander Fleming in 1928 (Fleming, 1929).

In the present scenario, antibiotics available in the market are either produced by microbial fermentation or are derived via semi-synthetic route using the existing antibiotic backbone structure. They are classified into different chemically defined groups. Antibiotics target bacterial physiology and biochemistry, causing microbial cell death or the cessation of growth. A significant number of these antibiotics affect cell walls or membranes (e.g., β-lactam and glycopeptides), while several others exert their antibacterial activity by targeting protein synthetic machinery via interaction with ribosomal subunits and these include antibiotics such as macrolides, chloramphenicol, tetracycline, linezolid, and aminoglycosides. Other “mechanistic” groups include molecules which interfere with the nucleic acid synthesis [e.g., fluoroquinolones (FQ) and rifampin], while some others exert their effects by interfering with the metabolic pathways (e.g., sulphonamides and folic acid analog) or by disruption of the bacterial membrane structure (e.g., polymyxins, daptomycin, and others).

A surge of discovery of several such antibacterial and antifungal antibiotics accompanied with a new generation of semi-synthetic drugs initially led to euphoria that any infectious disease could be successfully controlled using antibiotics. However, emergence and propagation of bacterial strains, resistant to almost all the therapeutically useful antibiotics during the past few decades revealed the limitation of the wonder drugs. Though imprudent and excessive use of antibiotics is highlighted as a major causative factor behind the setback, it is evident by this time that antibiotic resistance does not call for exposure of the organisms to antibiotics. It is also found that genes involved in the biosynthesis of antibiotics and antibiotic resistance evolved thousands of years before antibiotics were introduced into the clinical practice. Hence both antibiotic and its resistance determinants have some other role in bacterial physiology.

## ROLE OF ANTIBIOTICS IN NATURE

Antibiotics in the biosphere are produced by microorganisms as secondary metabolites at a concentration much lower than the therapeutic dose. Waksman was convinced that antibiotics play “no real part in modifying or influencing living processes that occur in nature” (Waksman, 1961) though there is evidence to the contrary (Gullberg et al., 2011). Antibiotics are produced in the late stages of microbial stationary growth phase, decoupled from the doubling time, implying that they are not indispensable for sustenance of life of the producer organism. However, it is also a fact that the production of an antibiotic is a multi-step process that involves a number of genes. Hence it does not seem tenable that such a complex anabolic process has been sustained through evolution without having any obvious purpose to serve. Recent studies reveal that antibiotics do have some specific effects on the natural milieu of the microbes while they assume an entirely different role as antibacterial agents in the dose used in therapeutics. This dual role of antibiotics remains a riddle. In the natural habitat, the diverse microbial communities exist as a multi-cellular network. They constitute a huge reservoir of different metabolic activities and have to evolve continuously to encounter the constant environmental threat and different selection pressure. Survival is most often a major challenge because of the limiting nutrients. Under nutrient starvation, the organisms start secreting secondary metabolites, which are a diverse array of low molecular weight organic molecules, known as the parvome (Davies, 2009). The total number of parvome detected so far is at least an order of magnitude larger than the number of bacteria in the biosphere. Bacteria of the phylum Actinobacteria (Ventura et al., 2007), a huge taxonomic group of diverse genera with a high genomic guanine–cytosine (GC) content, produce millions of complex bioactive small molecules. The ecological role of the majority of these small molecules in nature remains largely unknown. Amongst these molecules, only a fraction has been identified to have antibiotic activity and has been extensively studied only from the therapeutic point of view whereas the significance of antibiotics in nature still remains a mystery. The prevailing confusion about its role in nature has been dealt by Julian Davis in an article titled: “are antibiotics naturally antibiotic?” (Davies, 2006).

### ROLE OF ANTIBIOTICS IN INTERSPECIES COMPETITION

Antibiotics are critical to the producer organisms in the natural environment as they are needed both for survival and competitive advantage. Thus antibiotics are widely perceived as an arsenal of the producer microorganisms that they use against other naturally occurring cohabiting microorganisms and eliminate these competing bacteria for the purpose of “empire building” in the microbial community (Davies, 1990). The concept of the empire building role of antibiotics stemmed from the soil borne nature of the antibiotic-producing microorganisms. In certain instances like plant–microbe interactions, the antibiotic-producing organisms have indeed been found to secrete antibiotics to eliminate the competing bacteria in the vicinity. *Pseudomonas fluorescens* 2-79 (NRRL B-15132) colonizing the rhizosphere of wheat, secrete the antibiotic, phenazine which inhibits the growth of *Gaeumannomyces graminis* var. *tritici* thus suppressing a major root and crown disease of wheat and barley (Thomashow and Weller, 1988). Few other ecological examples of probable antibiotic function of these secondary metabolites in nature include the fungus-growing ants, which carry an antibiotic-producing actinomycete (*Pseudonocardia* sp.) on their cuticle specifically for biocontrol of the fungal garden parasite, *Escovopsis* sp. (Currie et al., 1999; Cafaro and Currie, 2005). Another example is the antibiotic-producing strain *Streptomyces diastatochromogenes* strain PonSSII controlling the growth of *Streptomyces scabies* strain RB4, the causative agent of potato scab (Neeno-Eckwall et al., 2001).

### ROLE OF ANTIBIOTICS AS SIGNALING MOLECULES

During primordial development, the cells instead of antagonizing each other remained in a communal harmony to share an evolutionary advantage and the symbiotic coexistence of microbes in biofilms, in lichens and in a metabolically quiescent state is well-documented (Davies, 2006). Chemical communication, an essential feature in such mixed populations, helps the microorganisms coordinate with each other in an orchestrated fashion. Probably the small molecule antibiotics act as a liaison in the communication between the microbes. It has been postulated that antibiotics in the early stages of biochemical evolution had some functions mediated by their interaction with primitive macromolecule receptors. Later on, many of these low molecular weight interacting partners were employed to antagonize the original receptor sites in macromolecular structure thus imparting the antibiosis property. Such a switchover in the role of the primordial effector molecule has been hypothesized in a schematic biochemical pathway by J. Davies (Davies, 1990). During the past seven decades, the scientists were mostly preoccupied with studying the antibiotic property of these small molecules because of its overwhelming success in pharmacotherapy. Only during the recent past antibiotics were implicated in several other physiological phenomena ranging from control of transcription and translation to growth of the producer organism in the microbial communities. From a broader perspective of evolutionary and ecological point of view, antibiotics, apart from having a growth inhibitory activity, appear to have a role in intra- and inter-domain communication in various ecosystems. Under normal physiological condition, microorganisms in their marine and soil habitats produce antibiotics at sub-minimum inhibitory concentration (sub-MIC). Despite having evidence that resistant bacteria representing a low fraction in a sample were enriched at sub-inhibitory concentrations of three clinically useful antibiotics (Gullberg et al., 2011), antibiotics are believed not to display any antibiotic activity at concentrations lower than 1/100 of the minimum inhibitory concentrations (Davies, 2006). Instead, many antibiotics are known to possess biological activities other than the inhibitory role at such a low concentration and have major effects on global transcription pattern. Antibiotics such as erythromycin (an inhibitor of translation) and rifampicin (an inhibitor of transcription), at low concentrations, could modulate global bacterial transcription pattern in a random promoter library construct of *Salmonella typhimurium* having *lux as the* reporter gene. In response to the low concentration of different antibiotics, a variety of promoters were activated, including those involved in virulence, metabolic, and adaptive functions. However, transcription was markedly reduced in antibiotic-resistant hosts but the mutants defective in stress responses such as *rec* and *lex* (SOS), *dnaJ* and Δ*dnaKJ* (heat shock response), and Δ*relA* Δ*spoT* (universal stress response) could not prevent antibiotic-induced modulation of transcription pattern at sub-inhibitory concentration (Goh et al., 2002). Antibiotics having different binding sites on the ribosome (chloramphenicol, aminoglycosides, macrolides, and tetracycline), or intruding into the process of cell-wall synthesis (some β-lactams and fosfomycin) were reported to alter the transcription pattern under different circumstances. So it has been postulated that the transcription machinery must be capable of sensing these subtle variations in the conformation or stoichiometry of small molecule antibiotics to respond to specific up- or downregulation.

These small molecules have a significant role in the dynamics of bacterial communities in nature, thus contributing to both competitive and interactive responses. Inhibition occurs when high concentrations are attained, transcriptional changes occur at low concentrations (Davies et al., 2006). This dose-dependent dual role, defined as hormesis, is not exclusively associated with antibiotics but also found in different other biomolecules (Davies et al., 2006; Calabrese, 2009). The dual role of antibiotics has been confirmed by the detection of a variety of bioactivities of several antibiotics. Some β-lactam antibiotics were shown to stimulate the glutamate transporter-1 (GLT1) both in cell culture and in animal models (Rothstein et al., 2005). Thiostrepton, a potent protein synthesis inhibitor which binds to 23S RNA, was found to act as a transcription inducer at a concentration lower than the inhibitory concentration (Murakami et al., 1989). Lincomycin, which terminates the peptide bond elongation, could stabilize certain mRNAs like the *bla* mRNA (Matsushita et al., 1989). Puromycin, in spite of having a chain termination activity in polypeptide synthesis, can affect nucleic acid synthesis, mediated by its aminonucleoside moiety (Yarmolinsky and Haba, 1959). Edeine, apart from its inhibitory role in protein synthesis, has specific effects on nucleic acid synthesis (Kurylo-Borowska and Szer, 1972). Many of the so-called antibiotics have been shown to have sex pheromone activity and found to stimulate bacterial conjugation by several orders of magnitude (Evans and Dyke, 1988). Sub-MICs of antibiotics are thus known to induce extensive transcriptional changes in bacteria (Tsui et al., 2004; Yim et al., 2006a). In the majority of cases the underlying mechanisms of the different ecological role of the antibiotics at sub-inhibitory concentration still remain to be elucidated.

A second level of signaling via antibiotics has also been documented in different organisms. Cationic peptides, which have a broad range of antimicrobial activities against most Gram-negative and Gram-positive bacteria, fungi, enveloped viruses, and eukaryotic parasites, also have a broad spectrum of pro-inflammatory and anti-inflammatory effects on the host immune system. Apart from their antimicrobial activity, these molecules also act as transcription modulators. Cecropin, an insect-derived cationic peptide, was shown to alter the transcription pattern of 26 genes when added to *Escherichia coli* (Hong et al., 2003). Some cationic peptides of microbial- and mammalian origin (polymyxins B and E, cattle indolicidin and human LL-37) were found to activate the two component *pmrA–pmrB* regulatory operon, which regulates the resistance to polymyxin B and cationic antimicrobial peptides in *Pseudomonas aeruginosa* (McPhee et al., 2003). Inhibitory effect of several antibiotics on sporulation of bacteria is well-known (Ochi and Freese, 1983; Babakhani et al., 2012).

### ROLE OF ANTIBIOTICS IN VIRULENCE

Some antibiotics, at concentrations below the MIC, are able to modulate the expression of some genes associated with virulence. Up- and downregulation of the transcription of virulence and motility genes were induced by the RNA polymerase inhibitor, rifampicin (Yim et al., 2006b). Sub-inhibitory concentration of several other antibiotics such as metronidazole, vancomycin, clindamycin, and linezolid could induce the early transcription of *tcdA* (*toxin A*) and *tcdB* (*toxin B*), the major virulence factors-encoding genes of *Clostridium difficile*, a nosocomial pathogen responsible for diarrhea, during exponential phase growth of the organism (Gerber et al., 2008). Imipenems, at sub-inhibitory concentration, were shown to induce β-lactamase and production of alginate during biofilm formation in *Pseudomonas aeruginosa*. Sub-inhibitory concentration of an aminoglycoside antibiotic tobramycin induced biofilm formation in *Pseudomonas aeruginosa* and *Escherichia coli *(Hoffman et al., 2005). On the other hand, sub-inhibitory level of the macrolide antibiotic azithromycin was shown to elicit the exactly opposite phenomenon like suppression of the alginate overproduction and biofilm formation in *Pseudomonas aeruginosa* (Ichimiya et al., 1996). These contradictory observations suggest that the molecular targets of different antibiotics for the execution of their inhibitory effects could be similar but they might differ extensively while functioning as signaling molecules (Aminov, 2009). This postulation is substantiated by the fact that tobramycin at sub-inhibitory concentrations does not bind the ribosome (the usual target for its role as an antibiotic), rather it targets the aminoglycoside response regulator gene (arr) which is responsible for the induction of biofilm-specific aminoglycoside resistance. The gene is also predicted to encode an inner-membrane phosphodiesterase acting on the substrate, cyclic di-guanosine monophosphate (c-di-GMP), a bacterial second messenger that regulates cell surface adhesiveness (Hoffman et al., 2005).

The influence of sub-inhibitory doses of several antibiotics on the expression of the gene encoding the *Staphylococcus aureus* alpha-toxin (*hla*), a major virulence factor of the pathogen, revealed that glycopeptide antibiotics had no effect whereas β-lactams induced a strong expression of *hla*. On the other hand, the macrolide erythromycin and several aminoglycosides reduced, while FQs slightly stimulated *hla* expression and an almost complete inhibition of alpha-toxin expression was obtained using clindamycin. Furthermore, methicillin enhanced Hla production of both MSSA (methicillin-sensitive *Staphylococcus aureus*) and MRSA (methicillin-resistant *Staphylococcus aureus*; Ohlsen et al., 1998). The sub-inhibitory concentration of ceftazidime (β-lactam) and ciprofloxacin (FQ) appeared to be effective in decreasing the expression of a range of quorum sensing (QS)-regulated virulence factors (Skindersoe et al., 2008).

Genomic rearrangements in pathogenic strains of *Escherichia coli* and *Shigella* following exposure to sub-inhibitory concentrations of kanamycin and ampicillin was demonstrated (Pedro et al., 2011). Some of the rearrangements may engender genotypes with increased virulence. SOS response, induced by ciprofloxacin, was found to stimulate transfer of pathogenicity Island in *Staphylococcus aureus* (Ubeda et al., 2005). Thus antibiotic used for the treatment of clinical infections may lead to the dissemination of virulence in some cases.

### ROLE OF ANTIBIOTICS IN HOST–PARASITE INTERACTION

Antibiotics have also been found to modulate host immune cell response, mostly mediated by cytokines. There are ample evidences of sub-inhibitory concentrations of antibiotics interference with processes of host–parasite interactions such as phagocytosis, adherence, and virulence. Antibiotics promote phagocytosis by altering the surface properties of pathogens. Many clinical isolates of *Enterococcus faecium* are resistant to neutrophil-mediated phagocytosis but exposure of vancomycin-susceptible *Enterococcus faecium* to quinupristin/dalfopristin at concentrations both at either sub-inhibitory or suprainhibitory concentrations promoted susceptibility to subsequent PMN (polymorphonuclear leucocytes)-mediated phagocytosis. Vancomycin-resistant strains on the contrary exhibited very little change in their binding toward PMNs after antibiotic pretreatment (Herrera-Insúa et al., 1997). Use of amoxicillin for the treatment of *Haemophilus influenzae* in acute otitis media caused upregulation in the expression of cytokines, interleukin (IL)-6, tumor necrosis factor-α, and IL-10 (Melhus, 2001).

### ROLE OF ANTIBIOTICS IN SOS AND DNA REPAIR GENE EXPRESSION

The SOS response, a regulatory network present in most bacteria is induced in response to DNA damage and has been shown to promote stress-induced mutagenesis, virulence, and dissemination of antibiotic resistance specifically by horizontal gene transfer (HGT). Beaber et al. (2004) showed that ciprofloxacin induced transfer of the *Vibrio cholerae* SXT integrative conjugative element (ICE) was controlled by SOS through RecA-dependent cleavage of the SXT encoded repressor SetR. SXT is a 100-kb ICE derived from *V. cholerae* that encodes genes for the resistance toward chloramphenicol, sulfamethoxazole, trimethoprim, and streptomycin (Beaber et al., 2004). Even the induction of the CTX prophage which encodes the cholera toxin is regulated by the SOS repressor LexA in conjunction with the phage repressor rstR which is also regulated by LexA (Quinones et al., 2005). In *V. cholerae*, the recombination-mediated excision of integron cassette was induced by SOS in response to different antibiotics (Guerin et al., 2009). On the other hand, SOS-independent recombination between divergent sequences either by RecBCD or RecFOR pathways was demonstrated in *Escherichia coli* by quinolones (DNA-damaging antibiotics) induced emergence of drug resistance (López et al., 2007).

Fluoroquinolones at sub-MIC have been shown to upregulate both the SOS and the methyl mismatch repair (MMR) pathway genes in *Staphylococcus aureus *with different SOS response and DNA repair-associated promoter-*lux* gene fusion construct (Mesak et al., 2008). Upregulation of several genes involved in the SOS response such as *umuD*, *lexA*, *sbmC*, and *dinP* was demonstrated in *Salmonella* using sub-inhibitory concentrations of quinolones. In addition, transcriptional regulators, genes putatively associated with membrane integrity (*spr*), virulence (*sicA*), and metabolism (*plsB*) were also affected (Yim et al., 2011). On the contrary, it has also been shown that exposure of *Streptococcus pneumoniae* to quinolones, aminoglycosides, and penicillin at sub-inhibitory concentrations may result in increased mutability, without the apparent involvement of SOS-response components (Henderson-Begg et al., 2006; Cortes et al., 2008).

SOS response also enhances frequency of mutations. Hence antibiotics which induce SOS response, contribute to the increase in the frequency of resistant strains. Exposure to ciprofloxacin was found to increase in the frequency of rifampicin-resistant mutants of *Streptococcus pneumoniae* almost by fivefold (Henderson-Begg et al., 2006). Carbapenem resistance in *Pseudomonas aeruginosa* was also found to be enhanced in presence of the same antibiotic almost in the same order (Tanimoto et al., 2008).

Thus low antibiotic concentration regulates the transcription of different genes in target bacteria while increasing higher concentration elicits a stress response and even higher concentration is lethal. Therefore, it is apparent that these small molecule at a sub-inhibitory concentration participate in a new form of signaling network by binding to different cytoplasmic macromolecular receptors such as ribosomes, and those involved in DNA replication, RNA transcription machinery, and cell-wall biosynthesis (Ryan and Dow, 2008). These receptors were also identified earlier as the inhibitory targets for the bioactive molecules at higher concentration. The cellular targets for the lethal and sub-inhibitory concentration sometimes also differ thus implying that antibiotics are multi-ligand-specific sensor molecules targeting different ligands in a concentration-dependent manner (Hoffman et al., 2005).

### ANTIBIOTICS AND QUORUM SENSING

Quorum sensing is a mode of bacterial communication in response to release of chemical messenger which regulate bacterial gene expression (Bassler, 1999). Secondary metabolites are activated during slow growth in the stationary phase and trigger the transition from primary to secondary metabolism (Bibb, 2005). This transition is a complex process and involves many signals, including those by small signaling molecules called γ-butyrolactones (also known as autoregulatory factor or A-factor). These signaling molecules, found mainly in *Streptomyces* species, are considered “bacterial hormones” or the QS factor. The QS signaling network initiates a set of metabolic changes leading to a number of events including morphological differentiation and synthesis of secondary metabolites such as antibiotics. The γ-butyrolactones constitute the major group of QS molecules in bacteria. The first γ-butyrolactone, A-factor, was identified in *Streptomyces griseus* in 1967 (Hsiao et al., 2009).

Biosynthesis of γ-butyrolactones is not well understood, but seems to be mediated by γ-butyrolactone synthase AfsA. The A-factor (2-isocapryloyl-3R-hydroxymethyl-γ-butyrolactones), at a certain critical concentration, binds to cytoplasmic receptor protein, a dimer of ArpA, which primarily acts as repressors by binding to the promoter region of adpA (A-factor-responsive transcriptional activator, a key pleiotropic regulator). The binding of the A factor dissociates ArpA from the promoter, thus facilitating the transcription and translation of adpA. Subsequently, AdpA binds an upstream activation sequence to initiate the transcription of strR (a pathway-specific regulatory gene responsible for transcription of other streptomycin biosynthetic genes cluster). strR is a pathway-specific regulator which acts as a transcriptional activator and induces transcription of most of the streptomycin biosynthetic genes by binding multiple sites in the gene cluster (Retzlaff and Distler, 1995). Thus onset of streptomycin biosynthesis is initiated by the QS factor, γ-butyrolactones. The gene *aph*D, encoding a major streptomycin resistance determinant streptomycin-6-phosphotransferase, is also under the control of the *adp*A-dependent promoter. The binding to γ-butyrolactones thus induces expression of the target genes (transcription factors) many of which are involved in regulation of specific antibiotic biosynthesis clusters. Onset of streptomycin biosynthesis is initiated by the QS factor, γ-butyrolactones (Retzlaff and Distler, 1995). The expression of the gene *aph*D, encoding a major streptomycin resistance determinant streptomycin-6-phosphotransferase, is also a QS-mediated event. Each receptor protein is highly specific for its cognate γ-butyrolactone. In *Erwinia carotovora* carbapenem biosynthesis is regulated by a classical autoinducer *N*-(3-oxohexanoyl)-L-homoserine. In another cephamycin C-producing bacterium, *Streptomyces clavuligerus*, antibiotic synthesis is regulated at the primary regulatory level by γ-butyrolactone (Liras et al., 2008).

Some 10–11 probable γ-butyrolactone synthates, all from the *Streptomyces* genus were found while 37–42 putative γ-butyrolactone receptors were found in the genome of different other bacteria apart from *Streptomyces*. Thus, it is believed that many of the cellular activities including the antibiotic production is under the control of a well-orchestrated QS signaling network mediated by a small population of γ-butyrolactone producers acting as a signaling factor for a diverse population of signal recipient (Aminov, 2009).

Structural similarity between antibiotic and intracellular signaling molecule has also been evidenced in several cases. The quinolones produced by *Pseudomonas aeruginosa* have a wide range of activities starting from antibiotics to autoinducers. 2-Heptyl-3-hydroxy-4-quinolone (pseudomonas quinolone signal; PQS), belonging to the family of 2-alkyl-4-quinolones (AQs), was previously described for their antimicrobial activities. Later on, it was found that PQS is integrated within an intricate QS circuit and plays an important role in *Pseudomonas aeruginosa* pathogenesis by regulating the production of diverse virulence factors including elastase, pyocyanin, and LecA lectin in addition to affecting biofilm formation (Dubern and Diggle, 2008; Heeb et al., 2011). Several other autoinducers like *N*-(3-oxododecanoyl) homoserine lactone and its tetrameric acid degradation product in *Pseudomonas aeruginosa* have antibacterial properties against Gram-positive bacteria (Kaufmann et al., 2005). *N*-(3-oxododecanoyl) homoserine lactone and other 3-oxo series homoserine lactones with 8, 10, and 14 length carbon chains were shown to inhibit growth of the Gram-positive *Staphylococcus aureus* (Qazi et al., 2006).

## ANTIBIOTIC RESISTANCE, THE TIP OF THE ICEBERG

In the late 1960s, the unprecedented successes of early antibiotic therapies led US Surgeon General William H. Stewart to make the famous declaration: “it is time to close the book on infectious diseases and declare the war against pestilence won” (Spellberg et al., 2008). The euphoria did not last long and the magic bullets started losing the efficacy because of the steady emergence of antibiotic-resistant pathogens simultaneously with their widespread use. Following the success of penicillin in controlling bacterial infection among the soldiers during the Second World War, emergence of penicillin-resistant strain was evidenced in the 1940s. By 1960, it assumed the shape of a pandemic problem. New β-lactam antibiotics were introduced into the clinical practice to restrain the problem. Simultaneously bacterial strains resistant to them came into being, a phenomenon dubbed as β-lactamase cycle. The first case of MRSA was identified in UK in 1961 (Johnson, 2011) and the first report on MRSA in the United States came in 1968. Now it is prevalent all over the world. MRSA is actually resistant to the entire class of penicillin-like antibiotics called β-lactams. Presence of an enzyme called New Delhi metallo-β-lactamase (NDM-1) in some Gram-negative bacteria (notably *Escherichia coli* and *Klebsiella pneumoniae*) makes them resistant to virtually all β-lactams including carbapenems, which are most often considered the last line of defense against multidrug-resistant pathogens (Kumarasamy et al., 2010). Isolation of bacterial strains producing extended spectrum β-lactamase (ESBL) resistant to third generation of cephalosporins and monobactams is also reported from time to time (Kurihara et al., 2013).

The glycopeptide antibiotic vancomycin was introduced in clinical practice in 1958 for the treatment of methicillin resistance in both *Staphylococcus aureus* and coagulase-negative staphylococci. Vancomycin resistance was so difficult to induce that it was believed to be very unlikely to occur in a clinical setting. Until the late 1980s, the glycopeptide antibiotic vancomycin was considered the drug of last resort for treatment of diseases caused by Gram-positive bacteria such as *Enterococcus faecalis*, MRSA, *Streptococcus pneumoniae*, and *Clostridium difficile* (Cunha, 1995). However, vancomycin resistance was first reported in coagulase-negative staphylococci in 1979 and 1983 (Srinivasan et al., 2002). At present vancomycin-resistant enterococci (VRE) pose a major challenge in therapeutics. Multidrug-resistant tuberculosis (caused by *Mycobacterium tuberculosis*) has assumed an epidemic proportion in some parts of the world. Multidrug-resistant strains of *M. tuberculosis*, now known to be present in 50 countries, heighten the threat posed by untreatable and fatal human tuberculosis.

Because of the rapid dissemination of antibiotic resistance in pathogens, many of the antibiotics, which were highly effective earlier, became obsolete during the past few decades. The efficacy of antibiotic treatment is on the wane as a result of the emergence and dissemination of antibiotic resistance amongst the pathogens. This warrants the discovery of newer and more promising antibiotics with long-term efficacy against various life threatening infections. However, the steady discovery of antibiotics witnessed during 1940–1980 could not be sustained. In the last two decades, only one new antibiotic class viz the oxazolidinone was introduced in the year 1990 and all other entrants were just the variation of the existing one (Raghunath, 2008). Eventually, clinical management of antibiotic-resistant superbugs is assuming the shape of a gruesome problem because of the depleting antibiotic reserve (Norrby et al., 2005). The widespread apprehension that we might be heading to a situation similar to the pre-antibiotic era cannot be dismissed as a far-stretched imagination.

Imprudent and excessive use of antibiotics is highlighted as a major causative factor behind the setback. The idea dubbed as Antibiotic Paradox by Levy (1992) is corroborated by numerous evidences. In 1967, the first penicillin-resistant *Streptococcus pneumonia* was observed in Australia, and after 7 years another case of penicillin-resistant *Streptococcus pneumonia* was reported from the U.S. in a patient with pneumococcal meningitis (Doern et al., 2001). In 1980, 3–5% of *Streptococcus pneumonia *was estimated to be penicillin-resistant and by 1998, 34% of the *Streptococcus pneumonia *sampled was resistant to penicillin (Doern et al., 2001). Antibiotic resistance in other organisms reflects the same trend as observed in *Streptococcus pneumonia *against penicillin. Tetracycline resistance by normal human intestinal flora exploded from 2% in the 1950s to 80% in the 1990s (Shoemaker et al., 2001). Kanamycin, an antibiotic used in the 1950s, has become clinically obsolete as a result of the prevalence of kanamycin-resistant bacteria (e.g., *M. tuberculosis*, *Campylobacter jejuni*).

On the other hand, it is also a fact that antibiotic resistance is observed even in bacteria isolated from totally uninhabited and thinly populated places, where they are unlikely or least likely to come in contact with the antibiotics (Chattopadhyay and Grossart, 2010). It is evident that antibiotic resistance is an outcome of evolution and pre-exposure of microorganisms to antibiotics is not a pre-requisite for emergence of resistance. In a systematic analysis, the conjugative plasmids occurring in several bacteria belonging to Enterobacteriaceae obtained from Murray collection were found to belong to the same incompatibility group as those present in contemporary Enterobacteriaceae (Datta and Hughes, 1983). It was shown that emergence and spread of conjugative plasmids, which are known to be a major vehicle for transmission of resistance, were not fostered by the widespread use of antibiotics, rather these plasmids evolved by inclusion of resistance-conferring genes in pre-existing plasmids (Datta and Hughes, 1983; Hughes and Datta, 1983).

Despite intensive investigations continuing for the last few decades in a global scale, no significant achievement has been made so far in the prevention and control of resistance development. A recent database reveals the existence of more than 20,000 potential resistance genes of nearly 400 different types in the available bacterial genome sequences. The fact that the number of functional resistance determinants in pathogens is much smaller (Liu and Pop, 2009) provides no relief since the new generation of therapeutically useful antibiotics are reaching the market only in trickle.

The World Health Organization (WHO) has long recognized anti microbial resistance (AMR) as a growing global health threat, and the World Health Assembly, through several resolutions over two decades, has called upon Member States and the international community to take measures to curtail the emergence and spread of AMR.

## EMERGENCE OF RESISTANCE PHENOTYPE

Antibiotic resistance is known to occur both in the pre-antibiotic and antibiotic-era. The pre-antibiotic era constitutes the time before the introduction of sulphonamides in 1930. It is well established that resistance phenotype and the antibiotics in the pre-antibiotic era coexisted in the natural environment without facilitating the process of selection of the deadly resistant pathogens.

The antibiotic era started following its discovery of antibiotics and their use in different spheres of life. The use of high concentration of lethal dose of antibiotics as a consequence of human activity led to a major change in innate functional role to give rise to the emergence of antibiotic-resistant pathogens within a short span of time. Antibiotic resistance genes, which were once involved in other cellular functions before human intervention, have been subsequently selected for the resistance phenotype with increased use of antibiotics. They have been mobilized from the environmental genomic reservoirs, with the rapid dissemination into taxonomically divergent commensal and pathogenic bacteria. Metagenomic approach in conjunction with a pipeline for the *de novo* assembly of short-read sequence data from functional selections (termed PARFuMS) has shown the evidence of lateral transmission of five different antibiotic resistance genes along with different non-coding region as well as multiple mobilization sequences from environmental reservoir of soil bacteria to the clinical pathogens (Forsberg et al., 2012). Thus HGT or lateral gene transfer, which contributes significantly to the evolution, maintenance, and transmission of virulence in pathogenic bacteria, also plays a pivotal role in dissemination of antibiotic resistance conferring genes from the environment in clinical settings (Colomer-Lluch et al., 2011).

Epigenetic inheritance-based evolution of antibiotic resistance genes has been reported in an isogenic population of *Escherichia coli*, exposed to gradually increasing concentration of different antibiotics such as ampicillin, nalidixic acid, and tetracycline. The high frequency of survival on low antibiotic concentration could not be accounted for by the occurrence of random spontaneous mutation. Instead it suggested that the antibiotic resistance genes were acquired by epigenetic inheritance. High reversion of this resistance phenotype further proved that it was indeed a case of epigenetic inheritance which does not impart a stable phenotype due to maintenance of certain chromatin configuration or DNA methylation state (Adam et al., 2008).

There are ample evidences to suggest that anthropogenic factors (use of antibiotics by human both for therapeutic and non-therapeutic purposes and disposal of the unused antibiotic formulations) inflict significant changes on the natural flora of bacteria. But the actual concentrations of antibiotics (and other pharmaceuticals) that bacteria in the natural population is exposed to, remain unknown in most of the cases (Kümmerer, 2003, 2004). Its role in the emergence of antibiotic resistance still remains highly controversial (Bhullar et al., 2012).

In therapeutics, the pathogens are challenged with an overwhelmingly high concentration of a single or few antibiotics whereas the soil borne microbes exist in a complex microenvironment and encounter a number of simultaneously occurring stress factors in a network of multiple interactions. Probably, these interactions have a nullifying effect on each other thus favoring the evolutionary selection of antibiotic sensitivity over resistance (Chait et al., 2011). Therefore, the dynamics of antibiotic resistance in clinical settings is believed to be inflicted by anthropocentric factors and is quite different from naturally occurring resistance. Simulation of these natural niches may reveal the dynamics by which antibiotic resistance is disseminated across the microbial population.

## IMPLICATION OF SUBLETHAL CONCENTRATIONS OF ANTIBIOTICS IN DISSEMINATION OF RESISTANCE

In nature, sub-inhibitory concentrations of antibiotics are probably encountered by bacteria more frequently than inhibitory concentrations. Most often bacteria are exposed to antibiotics at a sublethal concentration in certain clinical situations such as incomplete treatment of an infection, patient non-compliance, and reduced or limited drug accessibility to certain tissues (e.g., bone or cerebrospinal fluid; Bryskier, 2005). In the colon of a person taking antibiotics, sub-inhibitory concentrations would be experienced by colonic bacteria during the initial phase of treatment and at the end of treatment. Thus host tissues are exposed to a range of drug concentration starting from higher to a sub-inhibitory concentration. Therefore, micro-niches within the host, such as epidermis, lungs, and joints, may attain significantly lower drug concentrations than the plasma (Rybak, 2006). Outside clinical settings, sub-inhibitory condition is established because of the use of manure from livestock whose feed is supplemented with antibiotics and in consequence multiple drugs in a very low level find their way both to the soil and aqueous environment.

Due to such imprudent use of antibiotics, bacteria are quite likely to experience sublethal levels of antibiotics which have high implications in the spread of multidrug resistance. There is evidence that low level of antibiotics gives rise to mutagenesis in a wider range of antibacterial resistance genes and drug efflux systems resulting in multidrug resistance (Girgis et al., 2009). Sub-inhibitory concentrations of β-lactams were found to enhance the transfer of tetracycline resistance plasmids in *Staphylococcus aureus* by up to 1,000-fold (Barr et al., 1986). Ampicillin treatment of *Escherichia coli* was associated with the formation of norfloxacin-resistant isolates with mutations in *gyr*A, *gyr*B, or the *acr*AB promoter (PacrAB) and kanamycin-resistant isolates with mutations in *rpsL* or *arcA* (A and B). It has been hypothesized that low level of bactericidal antibiotics give rise to reactive oxygen species (ROS) which leads to DNA damage-induced mutations thus facilitating the emergence of multidrug-resistant bacteria (Kohanski et al., 2010). Induction of prophage in animal feed by antibiotics was evidenced. It may contribute to dissemination of antibiotic resistance (Allen et al., 2011). Induction of shigella toxin-encoding bacteriophage by ciprofloxacin and enhanced Shiga toxin (Stx) production from *Escherichia coli* O157:H7 was demonstrated *in vitro* and *in vivo*. Hence antibiotic-induction of phage may also contribute to increased virulence (Zhang et al., 2000).

In many cases, exposure to a low-dose antibiotic is associated with mobile element-mediated dissemination of antibiotic resistance genes through HGT which is known to be enhanced by sub-MIC concentrations of tetracycline (Celli and Trieu-Cuot, 1998). Tetracycline at a sub-inhibitory concentration in the mating medium substantially enhanced Tn916 mediated conjugal transfer to the recipient *Bacillus thuringiensis* subsp. *israelensis* (Showsh and Andrews, 1992). Spread of antibiotic resistance genes between human colonic *Bacteroides* spp. is mediated by self-transmissible elements known as conjugative transposons (CTns). The exposure of donor *Bacteroides* cells to low concentration of tetracycline appeared to be a pre-requisite for the excision of the CTnDOT family of CTns from the chromosome and conjugal transfer of the excised elements. Virtually no transfer occurs without the tetracycline induction of donor cells (Stevens et al., 1993; Whittle et al., 2002). Low concentrations of tetracycline might increase the likelihood of HGT of integrated mobile elements, such as CTnDOT and NBU1. At sub-inhibitory tetracycline concentration, the excision is not associated with growth phase (Song et al., 2005). Mobilization of coresident non-conjugative plasmids (from *Bacteroides* strain to *Bacteroides* strain or *Escherichia coli*) by chromosomally encoded tetracycline conjugal elements (Valentine et al., 1988) was enhanced by 20–10,000-folds when the donor was pre-grown in a sub-inhibitory concentration of tetracycline (1 μg/ml). The similar stimulatory effect of tetracycline on conjugation transfer was also demonstrated for the conjugative transposon Tn925 (Torres et al., 1991).

These *in vitro* observations were validated *in vivo* in gnotobiotic mice where tetracycline at a sublethal concentration in drinking water could induce the frequency of conjugative transfer of the transposon Tn1545 from *Enterococcus faecalis* to *Listeria monocytogenes* in the digestive tract and there was an approximately 10-fold increase in the transposition (Doucet-Populaire et al., 1991). In gnotobiotic rats, selection for the resistant phenotype was the major factor causing higher numbers of Tn916 transconjugants in the presence of tetracycline (Bahl et al., 2004). Therefore, the enhancement of conjugal transfer of antibiotic resistance-carrying transposons in the presence of sub-inhibitory concentration of antibiotics is not only an *in vitro* phenomenon but also takes place in the human intestinal microbiome of animal models.

Adverse effect of some antibiotics on bacterial motility was reported earlier (Molinari et al., 1992; Kawamura-Sato et al., 2000). However, sub-inhibitory concentration of tobramycin was observed to increase motility of *Pseudomonas aeruginosa* and enhance the expression of virulence determinants of the organism (Linares et al., 2006).

## ANTIBIOTIC RESISTANCE AND ITS ROLE IN NATURE

Antibiotic resistance can be defined in different ways: from the microbiological point of view, the resistance is defined as a phenotype which makes the microorganism less susceptible than other members of the same species irrespective of any level of resistance. On the contrary, when the resistance reaches a certain critical level so as to interfere with the pharmacotherapy of a clinical problem caused by the bacterium, it is called clinical resistance (Cantón and Morosini, 2011).

In the present scenario, acquired clinical resistance of pathogens is a major concern in the healthcare management which results from genetic changes involving mutation or HGT. Besides acquired resistance dealt with in this article, some other types of antibiotic resistances are also observed in bacteria. For example in some cases tolerance of a bacterium to a certain antibiotic is dependent on its metabolic state. It exhibits susceptibility to the antibiotic in growing state but become resistant to the same antibiotic in stationary phase. The micro-environments in a biofilm also contribute to the differential susceptibility of bacteria embedded in different parts of the polysaccharide matrix. Swarming, a phenomenon induced in some bacteria in response to nitrogen limitation, confers resistance to some antibiotics (Martinez and Rojo, 2011). Resistance to a variety of antibiotics was observed in swarming cells of some bacteria. This observation together with the resistance observed in biofilm clearly indicates that antibiotic resistance is a manifestation of social behavior of bacteria (Lai et al., 2009). Accumulation of the alarmone (p) ppGpp was also reported to modulate antibiotic resistance. Involvement of this alarmone in persistence (resistance occurring in a small fraction of naturally occurring bacteria) is postulated (Jayaraman, 2009). This type of resistance, known as phenotypic resistance, significantly contributes to some problems in clinical management of some bacterial infections.

The most ancient resistance also known as intrinsic resistance (Sheldon, 2005) is species-specific and results from impermeability of the cells to the antibiotic or lack of target of an antibiotic to a certain organism. However, besides these passive factors, intrinsic resistance is also caused by some antibiotic-detoxifying determinants encoding chromosomally encoded β-lactamases, efflux pumps or target-protecting proteins. This antibiotic resistance is widespread in nature and this huge intrinsic resistome of bacteria consists of genes of varied phylogenetic origin This intrinsic resistome has a high degree of non-specificity and many of the proteins encoded by these genes are involved in the basic physiological functions in the natural ecosystem as demonstrated in *Pseudomonas aeruginosa*, the novel nosocomial Gram-negative pathogen of environmental origin (Fajardo et al., 2008). The presence of antibiotics acts as a switch in physiological function to the antibiotic-resistance phenotype. These resistance genes persist in bacteria even in antibiotic-free environment thus further substantiating their alternative role in bacterial physiology. Tet(L) and Tet(K), the specific tetracycline c-resistance determinants are known to catalyze Na^+^ and K^+^ exchange for protons (Krulwich et al., 2001). Tet(L), a tetracycline-efflux transporter, and MdfA, a multidrug-efflux transporter, both responsible for alkali tolerance offer a striking example in support of this hypothesis (Krulwich et al., 2005). In *M. tuberculosis* the P55 efflux pump plays an important role in oxidative stress response and *in vitro* cell growth in addition to contributing to its intrinsic resistance phenotype (Ramón-García et al., 2009).

It is obvious that bacteria evolve with antibiotic resistance determinants get selective advantage over their antibiotic-sensitive counterparts in presence of antibiotics. However, some of the multidrug resistance determinants confer resistance not only to antibiotics but also to a number of structurally unrelated compounds viz, ethidium bromide, quaternary ammonium compounds, the DNA-intercalating mutagen acridine, the anionic detergent sodium dodecyl sulfate and uncouplers. Hence they appear to have a greater role in bacterial physiology. They also confer resistance to some chemical substances produced by the host, e.g., bile acids. It is speculated that antibiotic resistance is an offshoot of some hitherto undefined physiological roles played by the determinants in these bacteria (Martinez and Rojo, 2011).

Most antibiotic-producing strains carry genes encoding resistance to the antibiotics that they produce (Hopwood, 2007; Tahlan et al., 2007) and these genes are usually found in the same gene cluster as the antibiotic biosynthesis pathway genes (Benveniste and Davies, 1973; Martin and Liras, 1989). The proximity of the antibiotic resistance gene with the antibiotic biosynthetic gene as evidenced in *Streptomyces coelicolor* (where the genes encoding export proteins are embedded in the actinorhodin biosynthesis pathway) led to the postulation that these resistance determinants could have some role in the biosynthetic pathway of antibiotics (Allen et al., 2010). Antibiotic resistance genes are frequently present even in non-producer strains (Yamashita et al., 1985). In consequence, bacteria have evolved with a diverse pool of genes (the “resistome”) that protect them against the therapeutic dose of antibiotics. Gene orthologous to these have been identified on mobile genetic elements in resistant pathogens in clinical settings. These genes that make up this environmental resistome have the potential to be transferred to pathogens and indeed there is some evidence that at least some clinically relevant resistance genes have originated in environmental microbes (Wright, 2010).

## ANCIENT ORIGIN OF ANTIBIOTIC RESISTANCE GENES

It is well-established documented that the antibiotic resistance is a long-evolved trait in prokaryotes and the diverse pool of resistant genes co-evolved with antibiotics in non-clinical (natural) environment much before these have been used in human therapy. Most of the antibiotic producer organisms carry the respective antibiotic resistance genes and both the resistance and the antibiotic biosynthesis pathway genes are usually found in the same gene cluster (Allen et al., 2010).

In the evolutionary scale many antibiotics or their structurally related precursors are believed to be as old as amino acids. It has been suggested that antibiotics are more than 500 million years old, dating back to the Cambrian period and probably evolved at the same time vertebrate fish emerged (Baltz, 2008). The components of the antibiotics are believed to be even older and the postulation is substantiated by the occurrence of non-protein amino acids of peptide antibiotics in meteorites and other primordial sources (Johnson et al., 2008). Julian Davies at the University of British Columbia proposes that antibiotics could be some of the oldest biomolecules (Amábile-Cuevas, 2003).

Metagenomic analyses of ancient DNA from 30,000-year-old to Beringian permafrost sediments dating back to the late Pleistocene age demonstrated the presence of different antibiotic resistance genes encoding resistance to β-lactam, tetracycline, and glycopeptide antibiotics (D’Costa et al., 2011). Structural and functional studies of one of these ancient resistance genes, the *vanA* (vancomycin resistance operon) that encodes an ATP dependent D-alanyl-D-lactate ligase confirmed that it is essentially indistinguishable from VanA ligase associated with the recently discovered vancomycin resistance in the clinic.

Very recently, Lechuguilla Cave in New Mexico, cut off from the human activity for over four million years, was found to harbor culturable microbiomes, highly resistant to antibiotics. Some strains were found to be resistant to as many as 14 different commercially available antibiotics. Resistance was detected to a wide range of structurally different antibiotics including daptomycin, an antibiotic of last resort in the treatment of drug resistant Gram-positive pathogens. Enzyme-mediated mechanisms (e.g., glycosylation, phosphorylation) leading to resistance against both natural and semi-synthetic antibiotics were also discovered in some ancient bacteria. Characterization of macrolide kinase obtained from one of these resistant organisms revealed it to be related to a known family of kinases circulating in drug resistant pathogens at present (Bhullar et al., 2012).

In another study, molecular analysis of a metagenomic library from the cold-seep sediments of the deep sea Edison seamount (about 10,000 years old) also demonstrated the presence of TEM-type ESBLs (TEM-1 and TEM-116) suggesting that β-lactam resistance in microorganisms is likely to be present prior to the modern antibiotic era, and the diversity of TEM β-lactamases is not a recent phenomenon, rather it is the result of a very ancient evolution (Song et al., 2005). Therefore, despite the fact that increase in the use of an antibiotic is directly associated with increase in the frequency of resistant strains, it is obvious that exposure to antibiotics is not a pre-requisite for the emergence of resistance. Even the diversification of the antibiotic resistance appears to occur much before the human intervention as evidenced by the occurrence of the TEM-type ESBL in the 10,000-year-old sediments of the Edison seamount. But the prevalence of the resistance bacteria having TEM-1 and TEM-116 was as low as 0.3% (25 of 8,823) and 0.06% (5 of 8,823), respectively (Allen et al., 2009). Recent investigations revealed that the origin of both antibiotic resistance and the biosynthetic genes date back to the ancient period much before the human civilization began. Both antibiotics and the resistance phenotype existed together in nature for a long time.

There are many other cases of occurrence of antibiotic resistance genes in apparently antibiotic-free environments. The metagenomic studies of remote Alaskan soil revealed the presence of divergent β-lactamase genes and a bifunctional β-lactamase (Allen et al., 2009). These β-lactamases are more closely related to ancestral homologs compared to those isolated in clinical settings and are capable of conferring resistance on *Escherichia coli* despite the evolutionary distance. In another example of antibiotic resistance in environments not impacted by antibiotics, the phenotype of more than 60% of the Enterobacteriaceae isolates from a pristine freshwater environment was found to be multidrug-resistant. An example of this evidence exists in the two strains of *Citrobacter freundii* that were collected prior to the antibiotic era (the 1920s) which carried ampC β-lactamase genes. The encoded AmpC β-lactamases were as active as the recent plasmid borne AmpC β-lactamases (class C) that were found in the antibiotic era (Barlow and Hall, 2002). Phylogenetic analysis of the genetically diversified serine β-lactamases suggests an ancient root for the antibiotic resistance genes (Hall and Barlow, 2003).

Even the Murray collection (procured before and after the introduction of antibiotics, i.e., between 1917 and 1952) exhibited ampicillin and tetracycline resistance in 11 out of 433 enterobacterial strains and the resistance was non-conjugative. These studies reveal that the determinants of antibiotic resistance existed in nature much before the human intervention. Millions of years ago, antibiotics and antibiotic biosynthetic pathways evolved suggesting that both antibiotics and the resistance genes are very ancient (Wright and Poinar, 2012). The presence of multidrug-resistant organisms even in this pristine environment reinforces the idea that the antibiotic resistome is ancient and omnipresent in the microbial genome.

## CAN THE DOOMSDAY BE POSTPONED?

In keeping with a policy package recommended by WHO in 2002 emphasis was given on the availability of proper facilities to ensure rapid testing of antibiotic resistance, regular documentation and sharing of the surveillance data, uninterrupted access to quality medical service, regulation on the sale of antibiotics, development of new diagnostic tests, and novel antimicrobials. But the problem continues to be unabated till now (Leung et al., 2011).

Recently, scientists (Marraffini and Sontheimer, 2008) from the Northwestern University have discovered a specific DNA sequence, called a CRISPR (clustered, regularly interspaced, short palindromic repeat) locus, which confer prokaryotes a type of acquired immunity against the acquisition of resistance genes by encoding a sequence-specific defense mechanism against bacteriophages and thus conferring an immunity toward HGT. Members of recently potentially virulent strains of enterococcal have been shown to lack complete CRISPR loci. Expression of the CRISPR interference has also been found to prevent the transfer of antibiotic resistance and virulent genes, not only *in vitro*, but also *in vivo*, during a pneumococcal infection (Bikard et al., 2012). “If this mechanism could be manipulated in a clinical setting, it would provide a means to limit the spread of antibiotic resistance genes and virulence factors in *Staphylococcus* and other bacterial pathogens,” hopes Erik Sontheimer, Associate Professor at the Weinberg College of Arts and Sciences.

The new Eco-Evo drugs and strategies target prevention of the evolution and emergence of resistant bacteria in the environment. This might be achieved by using inhibitors against four P’s viz, penetration (by using 7-valent conjugated vaccine, PC7 to prevent colonization of resistant serotype in human), promiscuity (using broad host range conjugation inhibitor), plasticity (recombinase inhibitor), and persistence (decontamination of high risk clones or mobile genetic elements) of resistant bacteria. This strategy appears implementable in view of the fact that preventive measures recommended in various steps are mostly adoptable. However, their long-term impact on the environment warrants rigorous testing before implementation (Baquero et al., 2011). In order to combat the looming crisis, a number of different other approaches have been adopted. Pharmaceutical companies are tapping new sources (samples obtained from tropical rain forests, myxobacteria, marine bacteria, extremophilic bacteria) for novel antibiotics. Development of antimicrobials against bacterial molecules, which were not targeted earlier (e.g., bacteria DNA polymerase III, the cell division protein FtsZ, fibronectin binding proteins) is aimed at. Genes indispensable for survival of pathogens are shortlisted in search of new targets. In another strategy, molecular techniques are being used to clone the antibiotic biosynthetic genes cluster of a strain into a different strain, ultimately to get a hybrid molecule with antibiotic activity. In another approach called directed evolution, libraries are developed with randomly mixed and matched gene clusters obtained from different antibiotic producer strains. These libraries are transformed into protoplasts and recombinants are screened for improved spectrum of antibiotic activity. Large number of new compounds synthesized on a solid support, are screened for activity in another technique called combinatorial chemistry. These new strategies are expected to generate new compounds with novel antimicrobial activities (Strohl, 1997).

Several secondary approaches like reduction of antibiotic consumption, preservation of existing therapeutics, development of new antibiotics, and development of new strategies like sensitization of pathogens (Toney et al., 1998) have been adopted. The formulation, augmentin, a combination of β-lactamase inhibitor (clavulanic acid) and amoxicillin, is being successfully used against a variety of penicillin-resistant pathogens.

## CONCLUSION

It is apparent that antibiotics are endowed with multifaceted activities and the cell modulates their role. The dual nature of antibiotic acting both as signaling molecule as well as growth inhibitory molecule suggests that antibiotic resistance genes have to co-evolve with antibiotics to safe guard the producer organism from antibiotic threat.

While it is obvious that dissemination of resistance-conferring genes has been facilitated following introduction of antibiotics into the clinical practice, we are yet to get sufficient evidence to conclude that human intervention is the only contributing factor behind the spread of resistance. Until and unless we generate required amount of information to fill up the gap of knowledge in this aspect, we cannot expect to devise measures which would effectively arrest the progress of the crisis.

Antibiotic resistance is a complex and continually evolving problem. A vast body of information has been engendered during the past 70 years on the antibiotic resistance and its dissemination. Comparative genomics, molecular genetics, combinatorial chemistry, and structural biology are being applied to explore new antibiotics but still the struggle is on. Several secondary approaches like reduction of antibiotic consumption, preservation of existing therapeutics, development of new antibiotics, and development of new strategies like sensitization of antibiotic-resistant organisms using QS inhibitors have been adopted but without much success.

Relatively rare genes that happened to confer antibiotic resistance were once involved in other cellular functions, but were selected for the resistance phenotype and mobilized from the environmental genomic reservoirs, with the rapid dissemination into taxonomically divergent commensal and pathogenic bacteria. This process was very rapid on an evolutionary scale and HGT, mediated by mobile genetic elements, played a prominent role in it. So it would be wise to plan a strategy to control the process of HGT to control subsequent transfer of resistance genes to pathogens.

## Conflict of Interest Statement

The author declares that the research was conducted in the absence of any commercial or financial relationships that could be construed as a potential conflict of interest.
